# Mosquito larvicidal and antimicrobial activity of protein of *Solanum villosum *leaves

**DOI:** 10.1186/1472-6882-8-62

**Published:** 2008-12-06

**Authors:** Nandita Chowdhury, Subrata Laskar, Goutam Chandra

**Affiliations:** 1 Mosquito and Microbiology Research Units, Parasitology Laboratory, Department of Zoology, the University of Burdwan, Burdwan-713104, India; 2 Natural Products Laboratory, Department of Chemistry, the University of Burdwan, Burdwan-713104, India

## Abstract

**Background:**

Mosquitoes are associated with the transmission of malaria, dengue, Japanese encephalitis, filariasis and other viral diseases throughout the globe, apart from being a nuisance pest. Biological control alone or as a part of integrated vector management stands to be a better alternative to the chemical controls aimed against pest mosquitoes. At the same time it is necessary to control bacteria by synthetic or natural means (plant products). Hence the present study was designed to screen the effect of mosquito larvicidal and antimicrobial activitiy of protein isolated from matured leaves of *Solanum villosum *against mosquito immatures and some pathogenic bacteria.

**Methods:**

Aqueous solvent extract of fresh mature leaves of *S. villosum *was tested against 3rd instar larvae of *Anopheles stephensi*, *Culex quinquefasciatus *and *Stegomyia aegypti *mosquitoes and against four pathogenic bacteria. The protein fraction was isolated and tested for mosquitocidal and antibacterial activities. Amino acid analysis was performed on isolated protein using PICO.TAG amino acid system. SDS-PAGE was also done to detect the bands of amino acid on the basis of their molecular weights.

**Results:**

Proteins isolated from mature leaves of *S. villosum *were found to have larvicidal and antimicrobial properties. Analysis of the isolated protein identified fifteen amino acids of which eight were essential amino acids. SDS-PAGE detected seven bands corresponding to different molecular weights in the range of 69–109 KDa.

**Conclusion:**

Proteins of mature leaves of *S. villosum *exhibited moderate larvicidal and antimicrobial activities. The study provides considerable scope in exploiting local indigenous resources for isolation of antimicrobial and mosquito larvicidal proteins.

## Background

Most people consider mosquitoes as an annoyance; these tiny assassins have the potential and lethal capacity to kill more than a million victims a year around the world [1]. Mosquito borne diseases such as malaria, filariasis, dengue, yellow fever, encephalitis etc. are continuing to be major health problems for the people [2]. Pesticide exposure among humans has been linked to immune dysfunction, various forms of cancer and birth defects [3]. It is, therefore, necessary to identify a safe, eco-friendly alternate source of larvicide in order to reduce mosquito menace.

The problem of microbial resistance to pathogenic bacteria is growing and the outlook for the use of antimicrobial drugs in the future is still uncertain. Therefore, actions must be taken to reduce this problem, for example, to control the injudicious use of antibiotic, develop research to better understand the genetic mechanisms of resistance, and to continue studies to develop new drugs, either synthetic or natural [4].

*Solanum villosum *is an ayurvedic herb with multiple folk medicinal properties and used for swelling, sore eyes etc. This plant is easily available to the local people [5]. Leaves of this plant are also eaten as boiled salad and its orange berries are used as fruits [5]. It has already been found that chloroform: methanol (1:1) extracts of mature leaves and green berries of *S. villosum *have mosquito larvicidal activities [6,7]. But active principles for this bioactivity have not been determined.

The objectives of the present study were to:

i. isolate the protein fraction from matured leaves of *S. villosum*

ii. determine the efficacy, if any, of leaf protein in killing 3^rd ^instar mosquito larvae of 3 species namely, *Culex quinquefasciatus *Say, 1823, vector of filariasis; *Anopheles stephensi *Liston, 1901, vector of urban malaria and *Stegomyia *(*Aedes*)*aegypti *Linn, 1762, vector of dengue, dengue hemorrhagic fever, yellow fever etc.,

iii. to examine the antibacterial role, if any, of leaf protein against 4 pathogenic bacteria viz, *Staphylococcus aureus *MTCC 2940 and *Bacillus subtilis *MTCC 441 (Gram positive) and *Escherichia coli *MTCC 739, and *Pseudomonas aeruginosa *MTCC 2453 (Gram negative) and determination of Minimum Inhibitory concentration (MIC).

iv. to determine the chemical composition, homogeneity and molecular weight of protein from mature leaf of *S. villosum*.

## Methods

### General experimental Procedures

Mature leaves of *S. villosum *were collected from the outskirts of Burdwan (23°16' N, 87°54' E), WB, India from March 2006 to February 2007. Plant samples were identified by a plant taxonomist, Dr. G. G. Maity, Department of Botany, Kalyani University, Kalyani, West Bengal, India.

All chemical reagents used in this study were of analytical grade. Reagents for SDS-Polyacrylamide gel electrophoresis (SDS-PAGE) were purchased from Sigma Chemical Co. (St. Louis, MO, USA). Protein standard kit for PAGE was purchased from GENEI, Bangalore, India.

### Preparation and preservation of protein from decoction

5 g of dried decoction of mature leaves were soaked in phosphate buffer (pH 7.2) for over night and filtered through Whatman filter paper (No. 40). Leaf protein was extensively dialyzed for 48 hrs at 10°C against deionised distilled water and lyophilized. Dried protein was then kept in a freeze and stored at 5°C for *Bioassay*. Protein was bio assayed against laboratory-reared 3^rd ^instar larvae of *An. stephensi*, *Cx. quinquefasciatus *and *St. aegypti *mosquitoes. They were treated with different percent concentrations of protein solutions of the leaves, following the standard WHO larval susceptibility test method [8]. The tests were conducted at room temperature (27–30°C). Concentrations (0.03, 0.05% and 0.1%) of the extract in water were prepared fresh and used for the tests during mid-May to mid-June. Solutions of the extract were prepared in distilled water. At each of the given concentrations, three replicates comprising 10 larvae each were exposed. Results were scored after 24 h of continuous exposure to the test solution and expressed as per cent mortality. A control set was also prepared having the same larval density of each species in distilled water without the application of the protein sample.

### Antibacterial assay

Four bacterial strains were used for the study. Gram positive bacteria include *S. aureus *MTCC 2940 and *B. subtilis *MTCC 441 and Gram negative bacteria include *E. coli *MTCC 739 and *P. aeruginosa *MTCC 2453. All the tested strains were reference strains and were collected from the Microbiology Laboratory of Burdwan Medical College, Burdwan, India. The bacterial cultures were maintained in nutrient broth (Himedia, M002) at 37°C and maintained on nutrient agar (Himedia, MM012) slants at 4°C.

#### Disc diffusion method

Antibiogram was done by disc diffusion method [9,10] using protein and commonly used antibiotics. The test quantity of protein was dissolved in sterile water. The surfaces of media were inoculated with bacteria from a broth culture. High potency bio-discs (Himedia) were placed on the agar. After 18 h of incubation at a specific temperature [(30 ± 1) °C for *B. subtilis *and 37°C for *S. aureus, E. coli *and *P. aeruginosa*], the plates were examined and the diameters of the inhibition zones were measured to the nearest millimeter and compared against standard antibiotic amoxicillin. A control set was prepared with the DMSO in which no isolated protein/antibiotics were added.

### MIC value determination

The MIC was determined by macrobroth dilution [11] and agar well diffusion [12]. 100 μl volume of two-fold serial dilutions of extracts reconstituted in 5% DMSO was introduced into triplicate wells in Muller Hinton Agar plates (MHA) pre inoculated with test bacterial strains. The protein fraction was allowed to diffuse into the MHA at room temperature before incubation at 37°C for 18 h. The reconstituted extract was serially diluted two-fold in Muller Hinton Broth (MHB, Oxoid). Duplicate tubes of each dilution were inoculated with 5 × 10^5 ^cells (cfu) of the test bacterial strain and cultures incubated in a water bath at 37°C for 18 h. Two-fold serial dilution amoxycillin (μg/ml concentration) were included in each experiment as controls. The MIC was taken as the lowest concentration of protein fraction or drugs showing clear zone of inhibition in the agar well diffusion technique and as the highest dilution (least concentration) of protein fraction showing no detectable growth in macrobroth assay.

### Amino acid analysis

Amino acid analysis of the isolated protein was performed using PICO.TAG amino acid system according to PICO.TAG operation manual (Waters, USA). Dialysed and dried leaf protein (20 μg) of *S. villosum *was hydrolyzed by 6 N HCl containing 5% thioglycollic acid [13] for 24 h at 105°C in the PICO.TAG workstation. Hydrolysed sample and standard amino acid mixture, standard A (0.005 ml) was taken in respective tubes (vials) and was dried completely. These were then derivatized [14] by phenyl isothiocyanate (PITC) solution (ethanol: triethyl acetate: water: PITC: 7:1:1:1 by volume) for 20 min at 25°C in a Nitrogen atmosphere. The vials were then dried and the samples were reconstituted in a diluent solution (Na_2_HPO_4_, 0.071% w/v in distilled water, pH 7.4; pH was adjusted by 10% H_3_PO_4 _containing 5% v/v acetonitrile. The samples were analyzed by HPLC at 38°C as per the PICO.TAG manual using a PICO.TAG C_18 _hydrophobic column (5 μm, 3.9 × 150 mm, waters and detection at 254 nm. Amino acids present in the unknown sample were determined quantitatively by comparing the peak areas (745 B data module print out) of amino acids present in standard A.

### SDS-PAGE electroporesis

SDS-Polyacrylamide gel electrophoresis (SDS-PAGE) was performed according to the method of Laemmli, 1970 [15]. It was carried out on Bio-Rad gels composed of stacking gel (5% w/v) using 1.0 M Tris-glycine buffer containing 0.4% SDS at pH 6.8 and resolving gel (12%, w/v) using 1.5 M Tris-glycine buffer containing 0.4% SDS at pH 8.8. Protein sample was dissolved in phosphate buffer (5 mg/ml) and mixed with a solubilization buffer Tris – HCl 6.22 mμ (pH 6.8) which contains 2% (w/v) SDS, 50% glycerol, a pinch of bromophenol blue and reduced with 0.9 mμ 2-mercaptoethanol in boiling water for 3 min. Protein sample was loaded onto each well and electrophoresis (Bio-Rad electrophoresis apparatus, Bio-Rad Laboratories, Hercules, CA) was conducted at constant current of 60 volts by a Bio-Rad electrophoresis constant power supply unit (Model 200/2, Bio-Rad Laboratories, 2000 Alfred Nobel Drive, Hercules, CA). After electrophoresis, gels were stained with 0.2% (w/v) AgNO_3 _solution after being treated with fixing solution (methanol-acetic acid-H_2_O-p-formaldehyde) and sodium thiosulphate solution. It was then treated with developer (Na_2_CO_3_-sodium thiosulphate-37% p-formaldehyde) until the bands came out. The gels were soaked with stop solution and stored in 30% methanol (v/v) at 4°C. Molecular masses were determined using the molecular weight standard kit from GENEI, Bangalore, India. The result was placed in Table 6.

### Statistical Analysis

Mortality rates were corrected with Abbott's correction formula [16]. The data obtained were subjected to Probit analysis, to calculate the median lethal concentration LC_50 _and LC_90 _value [17]. Since the readings of control (distilled water) experiments *in vitro*, antibacterial studies against those bacteria were zero, the data were analyzed by simple arithmetic means of the different extracts and the standard errors were compared with the control.

## Results and discussion

The protein extracted from mature leaves was tested for mosquito larvicidal activity against third instar larvae of *Cx. quinquefasciatus, An. stephensi *and *St. aegypti *and recorded LC_50_/LC_90 _values are presented in Table 1. No mortality was recorded in the control set.

**Table 1:** Bio assay test of the protein extract of mature leaves of *Solanum villosum *against third instar larvae of three mosquito species

**Ingredient**	**Type of mosquito larvae**	**Period of bioassay**	**LC_50_(ppm) (LCL-UCL)**	**LC_90_(ppm) (LCL-UCL)**
Protein	*An. stephensi*	24 h	644.745 (622.00–656.73)	1882.42 (1878.76–1898.04)
	*Cx. quinquefasciatus*	24 h	645.75 (640.21–700.34)	1890.67 (1852.00–2003.84)
	*St. aegypti*	24 h	747.22 (721.43–752.38)	2220.01 (1997.75–2249.86)

Antibacterial activity of mature leaf protein of *S. villosum *against *E. coli, P. aeruginosa, B. subtilis *and *S. aureus *are shown in Table 2 and the MIC values are presented in Table 3. The antibacterial efficacy of the protein was found to be moderate and more or less same against all the bacterial strains mentioned irrespective of gram positive or gram negative. No antibacterial activity was noticed in the control set. It is previously reported that Gram-positive bacteria are susceptible to extracts of related plants [18]. Gram negative bacteria, in general, are more resistant to plant extracts than the Gram positive bacteria and such resistance could be due to the permeability barrier provided by the cell wall or to the membrane accumulation mechanism [19]. Results of the present study are not in agreement with those previous studies [20,21].

**Table 2:** Antibacterial activity of specific concentration of protein extract of mature leaves of *Solanum villosum *compared to control by disc diffusion method

		**Antibacterial activity**			
		
**Medicinal plants**	**Concentration/disc**	***S. aureus***	***B. subtilis***	***E. coli***	***P. aeruginosa***
Protein	60 mg	15.0 ± 0.577	12.0 ± 0.23	15.2 ± 0.05	14.0 ± 1.577
Sterile water	-	00.0 ± 0.00	00.0 ± 0.00	00.0 ± 0.00	00.0 ± 0.00
Amoxycillin	30 μg	00.0 ± 0.00	00.0 ± 0.00	7.00 ± 0.05	14.0 ± 1.027

**Table 3:** Minimum inhibitory concentration of protein extract of mature leaves of *Solanum villosum *by serial dilution method

	**Antibacterial activity (MIC)**			
	
**Medicinal plants**	***S. aureus***	***B. subtilis***	***E. coli***	***P. aeruginosa***
Protein	1625 μg/ml	1900 μg/ml	1500 μg/ml	1800 μg/ml

**Figure 1:**
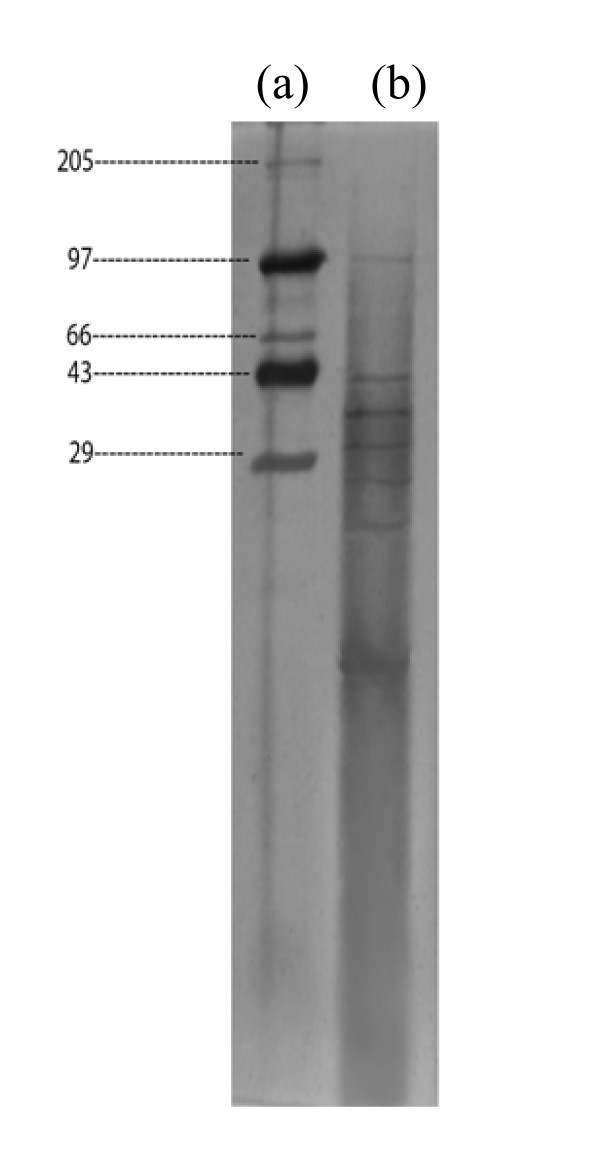
**PAGE of *Solanum villosum *mature leaf proteins.** The separations are as follows: (a) molecular weight marker-showing molecular weights of kD (b) proteins of plant tested.

Amino acid analysis of isolated protein from mature leaves of *S. villosum *revealed the presence of fifteen amino acids of which eight were essential amino acids (Table 4). Isolated protein was rich in aspartic acid, glycine, threonine and serine, but low in cystine (Table 4). Seven distinct bands were found from SDS-PAGE electrophoresis of the isolated proteins (Fig. 1) from the mature leaves of *S. villosum *corresponding to the molecular weights 109, 50, 38, 31.6, 24, 18.6 and 6.9 KDa (Table 5).

**Table 4:** Amino acid composition of protein of mature leaves of *Solanum villosum*

**Name of amino acids**	**g/16 g N**
Aspartic acid+ Asparagine	32.99
Glutamic acid+ Glutamine	3.46
Serine	6.29
Glycine	8.89
Histidine	----
Arginine*	5.74
Threonine*	9.14
Alanine	5.66
Proline	----
Tyrosine	5.02
Valine*	5.64
Methionine*	1.90
Cystine	1.44
Isoleucine*	4.78
Leucine*	4.00
Phenylalanine*	3.43
Lysine*	1.62
Total	100.00

* Essential amino acids.

^a^Mean of three results.

**Table 5:** Molecular weight determination of isolated proteins from the mature leaves of *Solanum villosum *by SDS-PAGE.

Proteins	Mobility	Mol. wt. from the literature (in Daltons)	Mol. wt. from a figure log mol wt. vs. mobility (in Daltons)
Standard Proteins:			
Std – 1	0.04	205,000	-
Std – 2	0.14	97,000	-
Std – 3	0.215	66,000	-
Std – 4	0.26	43,123	-
Std – 5	0.34	29,123	-

*Solanum villosum *proteins			

SV-1	0.14	-	109,000
SV-2	0.26	-	50,000
SV-3	0.30	-	38,000
SV-4	0.33	-	31,600
SV-5	0.37	-	24,000
SV-6	0.41	-	18,600
SV-7	0.56	-	6,900

Recently, much attention has been directed toward extracts and biologically active compounds isolated from popular plant species. The use of medicinal plants plays a vital role in covering the basic health needs in developing countries, and these plants may offer a new source of antibacterial and larvicidal products. The literature indicates that the biological activity is due to different chemical agents in the extract, including essential oils, steroids, alkaloids, flavonoids, triterpenoids and phenolic compounds or free hydroxyl groups. Antibacterial role of seed protein [22] and antibacterial and antifungal roles of leaf protein [23] has been documented previously but the role of plant protein as mosquito larvicide has been presented for the first time in this article. However future studies on the effect of this protein on non-target organisms, their mode of action and field trials are needed to recommend the isolated protein of *S. villosum *as a mosquitocidal and antibacterial product.

## Conclusion

Isolated proteins from mature leaves of *S. villosum *exhibited moderate larvicidal and antimicrobial activities during the present study. This study also provides considerable scope in exploiting local indigenous resources for further isolation of antimicrobial and mosquito larvicidal proteins.

## Competing interests

The authors declare that they have no competing interests.

## Authors' contributions

NC carried out the laboratory bioassay experimentation. SL helped in interpretation of data. GC participated in the conception, design of experiments, critical revision of the manuscript and coordination. All authors read and approved the final manuscript.

## Pre-publication history

The pre-publication history for this paper can be accessed here:



## References

[B1] Vatandoost H, Vaziri M (2001). Larvicidal activity of neem extract (*Azadirachta indica *2001.) against mosquito larvae in Iran. Pestol.

[B2] Das MK, Ansari MA (2003). Evaluation of repellent action of *Cymbopogan martinii martinii *2003. Stapf var *sofia *oil against *Anopheles sundaicus *in tribal villages of Car Nicobar Island, Andaman Nicobar Islands, India. J Vector Borne Dis.

[B3] Nigam SK, Venkatakrishna, Bhatt H (2001). Occupational cancer: Introduction and intervention. Ind J Occup Hlth.

[B4] Nascimento GGF, Locatelli J, Freitas PC, Silva GL (2000). Antibacterial activity of plant extracts and phytochemicals on antibiotic resistant bacteria. Braz J of Microbiol.

[B5] Edmonds JM, Chweya JA (1997). Promoting the conservation and use of underutilized and neglected crop; black night sheds (*Solanum nigrum *L.) and related species. International Plant Genetic Resources Institute Rome, Italy.

[B6] Chowdhury N, Bhattacharjee I, Laskar S, Chandra G (2007). Efficacy of *Solanum villosum *Mill. (Solanaceae: Solanales) as biocontrol agent against fourth instar larvae of *Culex quinquefasciatus *Say. Turk J Zool.

[B7] Chowdhury N, Ghosh A, Chandra G (2008). Mosquito larvicidal activities of Solanum villosum berry extract against the dengue vector *Stegomyia aegypti*. BMC Compl Alt Med.

[B8] World Health Organization (1981). Instructions for determining the susceptibility or resistance of mosquito larvae to insecticides.

[B9] National Committee for Clinical Laboratory Standards (1993). Performance standards for antimicrobial disc susceptibility tests.

[B10] Bauer AW, Kirby WM, Sheris JC, Turck M (1966). Antibiotic susceptibility testing by a standardized single disc method. Am J Clin Path.

[B11] National Committee for Clinical Laboratory Standards (NCCLS) (1993). Methods for dilution in antimicrobial susceptioblity tests: Approved standard M2-A5.

[B12] Okeke MI, Iroegbu CU, Eze EN, Okoli AS, Esimene CO (2001). Evaluation at extracts of the rest of Landolphia owerrience for antibacterial activity. J Ethnopharmacology.

[B13] Mastubara H, Sasaki RM (1969). High recovery of tryptophan from acid hydrolysis of proteins. Biochem Biophys Res Commun.

[B14] Ghosh AK, Naskar AK, Sengupta S (1997). Characterization of a xylanolytic amyloglucosidase of *Termitomyces clypeatus*. Biochem et Biophys Acta.

[B15] Laemmeli UK (1970). Cleavage of saturated proteins during the assembly of the read of bacteriophage 4. Nature.

[B16] Abbott WS (1987). A method of computing the effectiveness of an insecticide. 1925.. J Am Mosq Control Assoc.

[B17] Finney DJ (1971). Probit analysis.

[B18] Tkachenko FP, Koval VT (1990). Biochemical composition of abundant benthic seaweeds of the Black Sea. J Hydrobiol.

[B19] Cowan MM (1999). Plant product as antimicrobial agents. Clin Microbiol Rev.

[B20] Adwan K, AbuHasan N (1998). Gentamicin resistance in clinical strains of Enterobacteriaceae associated with reduced gentamicin uptake. Folia Microbiol.

[B21] Talas-Oğraş T, İpekçi Z, Bajroviç K, Gözükirmizi N (2005). Antibacterial activity of seed proteins of *Robinia pseudoacacia*. Fitoterapia.

[B22] Umar Dahot M (1988). Antimicrobial activity of small protein of *Moringa oleifera *1998. leaves. J Islamic Acad Sci.

[B23] Terras FRG, Eggermont K, Kovaleva V, Raikhel NV, Osborn RW, Kester A, Rees SB, Torrekens S, Leuven FV, Vanderieyden J, Cammue BPA, Broekaert WF (1995). Small cysteine rich antifungal proteins from radish: Their role in host defence. The Plant Cell.

